# Glucose Response during the Night Is Suppressed by Wheat Albumin in Healthy Participants: A Randomized Controlled Trial

**DOI:** 10.3390/nu11010187

**Published:** 2019-01-17

**Authors:** Shinichiro Saito, Sachiko Oishi, Aiko Shudo, Yoko Sugiura, Koichi Yasunaga

**Affiliations:** 1 Biological Research Laboratories, Kao Corporation, 2-1-3 Bunka Sumida-ku, Tokyo 131-8501, Japan; oishi.sachiko@kao.com; 2 Health Care Food Research Laboratories, Kao Corporation, 2-1-3 Bunka Sumida-ku, Tokyo 131-8501, Japan; shudou.aiko@kao.com (A.S.); sugiura.yoko@kao.com (Y.S.); yasunaga.kouichi@kao.com (K.Y.)

**Keywords:** glucose, human, night, postprandial, wheat albumin

## Abstract

Postprandial blood glucose excursions are important for achieving optimal glycemic control. In normal-weight individuals, glucose tolerance is diminished in the evening compared to glucose tolerance in the morning. Wheat albumin (WA) has the potential to suppress the postprandial glucose response with a relatively small dose, compared to the dose required when using dietary fiber. In the present study, the effect of WA on glycemic control during the night was investigated after a late evening meal. A randomly assigned crossover trial involving a single oral ingestion in healthy male participants was performed in a double-blind placebo-controlled manner. The participants ingested the placebo (PL) tablets or the WA (1.5 g)-containing tablets 3 min before an evening meal at 22:00 hour, and blood samples were drawn during the night until 07:00 hour using an intravenous cannula. The participants slept from 00:30 hour to 06:30 hour. Glucose response, as a primary outcome during the night, was suppressed significantly by the WA treatment compared to the PL treatment, but the insulin response was not. Plasma glucose-dependent insulinotropic polypeptide concentration during the night was lowered significantly by the WA treatment compared to the PL treatment. In conclusion, WA may be a useful food constituent for glycemic control during the night.

## 1. Introduction

An estimated 425 million people worldwide had diabetes in 2017, and this number is projected to reach 700 million by 2045 [1]. Many epidemiologic studies have demonstrated a complex association between glycemia and cardiovascular risk [2,3], with evidence suggesting that an acute increase in glycemia, particularly after a meal, may have direct detrimental effects on the cardiovascular system [4]. Until recently, there has been a strong emphasis on fasting plasma glucose, and the predominant focus of therapy has been on lowering hemoglobin A1c (HbA1c) levels [5]. Although the control of fasting hyperglycemia is necessary, it is usually not sufficient to achieve optimal glycemic control. A growing body of evidence suggests that the reduction of postprandial plasma glucose excursions is as important, or perhaps even more important for achieving HbA1c goals [6,7]. The use of a variety of both non-pharmacologic and pharmacologic therapies is recommended to control postprandial plasma glucose [7]. This is particularly relevant during the night, as glucose tolerance is diminished compared to its level in the morning, even in normal-weight individuals [8,9,10,11,12,13,14,15,16]. Therefore, even in healthy, non-diabetic people, the use of non-pharmacologic therapies, such as the control of dietary and fitness habits, to protect against impaired glucose tolerance during the night is recommended. 

Wheat albumin (WA) has a long history of consumption in humans as a natural food constituent and is a potentially protective agent against postprandial hyperglycemia via its alpha-amylase inhibiting activity with no change in insulin secretion [17], suggesting that WA might improve postprandial insulin sensitivity. Inhibitors of carbohydrate digestion and absorption have been reported to improve blood glucose control with a low risk of hypoglycemia [18], so WA might be a good candidate for night care. Additionally, WA has the potential to suppress the glucose response at a relatively lower dose [17] than other food constituents like wheat or oat fiber (~6 g) [19,20]. Therefore, it could be used in a wide variety of functional food products or incorporated easily into a habitual diet.

Thus, the primary objective of the present study was to investigate the potential effect of WA as a dietary therapy agent to protect against the diminished glucose response during the night in healthy individuals.

## 2. Materials and Methods

### 2.1. Ethics Approval and Consent to Participate

This study was performed in accordance with the tenets of the Declaration of Helsinki (2013) and was approved by the Ethical Committee of the Oriental Ueno Kenshin Center (Tokyo, Japan). After receiving a full explanation of the study, all participants provided written informed consent. The study was registered with the University Hospital Medical Information Network (UMIN) clinical registry, prior to the enrollment of the first participant, as UMIN000014533 (registered 15 July 2014 [21].

### 2.2. Study Design

**Figure 1:**
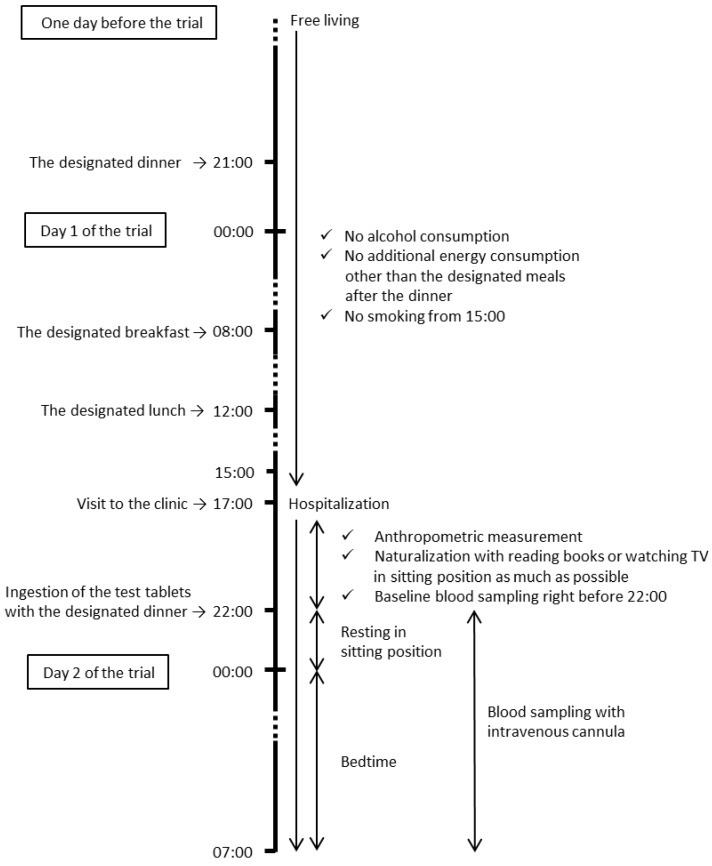
The study protocol.

This was a randomized, double-blind, placebo-controlled, crossover trial with a 1-week washout period, performed under the supervision of a physician in charge. Between the screening and the second visit, the participants were instructed to maintain and record their normal level of physical activity and their normal dietary, alcohol, and smoking habits. As shown in Figure 1, during the study, the participants were free-living, but were prohibited from drinking alcohol the day before the visits to the clinic and from smoking cigarettes for 2 hours before the visits. The participants ate designated meals for dinner in the evening at 21:00 hour one day before the trial, and for breakfast at 08:00 hour and lunch at 12:00 hour on the day of the trial. The participants were not allowed any energy intake after the designated dinner until the trial. The participants visited the clinic at 17:00 hour and were examined by the physician in charge. From 17:00 hour to 22:00 hour, anthropometric parameters were measured, and for the rest of the time participants read books or watched TV for naturalization in a sitting position with no energy consumption. Immediately after obtaining a blood sample, the participants orally ingested a single dose of WA (1.5 g)-containing tablets or placebo (PL) tablets 3 min before ingesting a designated evening meal at 22:00 hour. Blood samples were then drawn every 30 min until 00:00 hour, and then at 01:00 hour, 02:00 hour, and 07:00 hour, using an intravenous cannula. The participants slept from 00:30 hour to 06:30 hour, and their sleep quality was measured using an ActiGraph (ActiGraph, Pensacola, FL, USA). The amount and timing of water consumption during the visits were controlled. The study was conducted at Sumida Hospital, Tokyo, Japan and managed by TES Holdings Co., Ltd. (Tokyo, Japan), a contract research organization (CRO). The CRO managed the random allocation, enrollment, assignment of participants, and blinding of the assignment, and assessed the outcomes under the supervision of the physician in charge. Throughout the study (from screening to finalizing the dataset), the treatment allocation was concealed from the people involved, including the participants, the caregivers, the physicians, the CRO members, the manufacturer of the test tablets, the person in charge of the allocation, and the outcome assessors.

### 2.3. Participants

The appropriate sample size for the primary outcome of postprandial blood glucose was estimated to be 20 participants based on the outcomes of our unpublished pilot study (power 0.8 and type I error 0.05). In the present study, potential participants were screened based on the following inclusion criteria: (1) 5.3 ≤ fasting blood glucose < 7.0 mmol/L, (2) 5.2 ≤ HbA1c < 6.5%, (3) 23 ≤ body mass index (BMI) < 30, and (4) 30 ≤ age < 60 years. Participants were excluded if they met the following exclusion criteria: (1) presence of liver, kidney, or heart disease; respiratory, endocrine, or nervous system disorder; metabolism or consciousness dysfunction; diabetes; or other disease, (2) surgery in the 2 months before the trial, (3) history of gastrectomy or enterectomy, (4) taking medications for hyperglycemia, lipidemia, or hypertension, (5) taking supplements or food for a specific health use authorized by the government, (6) allergies to any constituents in the test meal or tablets, (7) an unpleasant feeling during blood draws, (8) donated 200 mL or more of blood in the month before the trial, (9) habitual breakfast skippers, (10) heavy smokers (>20 cigarettes/day), or (11) shift workers. The conditions and procedures of the trial were reviewed with all participants before they signed the informed consent form. The participants were randomly assigned to each sequence (ingestion order) with stratified randomization for glucose, hemoglobin A1c, age, and BMI using computer-generated random numbers under blind conditions.

### 2.4. Test Tablets and Meals

The designated 3 meals (the dinner on the day before the trial, and the breakfast and lunch on the day of the trial) consisted of a Japanese-style diet, such as rice, miso soup, simmered vegetables, grilled meats, and snacks with a total of 9142 kJ (protein = 14%, fat = 22%, and carbohydrate = 64% of the total energy), were provided by the physician in charge before the trial. The test tablets contained 1.5 g WA in 3 tablets for a single oral administration. The PL tablets were prepared using identical ingredients, including flavors and preservatives, but did not contain WA. Each tablet weighed 1.1 g. The energy values were 14.7 kJ per WA tablet and 10.9 kJ per PL tablet. The tablets could not be distinguished by appearance, taste, or odor, and were provided to the CRO after concealment by the manufacturer. The CRO then re-concealed the test tablets and provided them to the participants. The test evening meal on the trial day consisted of curry and rice with a total of 2579 kJ (protein = 7%, fat = 11%, and carbohydrate = 81% of the total energy). 

### 2.5. Laboratory Measurements

The blood samples collected for measuring glucose, insulin, and triglyceride were centrifuged at 1000× *g* for 15 min at 4 °C to isolate the serum or plasma. The measurements were performed by the Health Sciences Research Institute, Inc. (Yokohama, Kanagawa, Japan). Blood samples for total glucose-dependent insulinotropic polypeptide (GIP) and active glucagon-like peptide-1 (GLP-1) were collected into BD P800 tubes (Becton, Dickinson and Company, Franklin Lakes, NJ, USA) on ice and centrifuged at 1000× *g* for 15 min at 4 °C to isolate the plasma. Total GIP and active GLP-1 were measured using commercially available enzyme-linked immunosorbent assay kits obtained from Immuno-Biological Laboratories, Co., Ltd. (Fujioka, Gunma, Japan). 

### 2.6. Statistics

The primary outcome of this study was the difference in the area under the curve (AUC) of the blood glucose response during the night (9 hours from 22:00 hour to 07:00 hour) between the treatments assessed with the mixed model, adjusted by sequences and treatments as a fixed effect. As an exploratory assessment, analysis of covariance was also performed on the slope of the changes in the parameters using the linear mixed model, and the *p*-values for the time and treatment effect and the treatment by time interaction were obtained. In addition, the Bonferroni correction for multiple comparisons was applied to assess statistical difference on each time point between the treatments. A two-sided *p*-value ≤ 0.05 was considered to indicate statistical significance. All statistical analyses were performed using IBM SPSS Statistics version 19 (IBM Co., Armonk, NY, USA).

## 3. Results

### 3.1. Characteristics of the Participants

Eighty-five individuals were screened, and 22 were recruited. Of those recruited, one participant missed the second visit for unknown reasons, and one dropped out due to feeling sick before ingesting the test tablets on the day of the second visit. Based on an examination by the physician in charge, there was no relationship between the reason for dropping out and the test tablets. Thus, 20 participants completed the study and were included in the analyses. The baseline characteristics of these participants are presented in Table 1. From screening until the second visit, no considerable habitual changes were recorded. The percentage of sleep did not differ significantly between the treatments (88.1 ± 2.3% after PL treatment and 86.3 ± 3.0% after WA treatment).

### 3.2. Effects of WA on Blood Glucose, Insulin, and Triglyceride Response during Night

**Figure 2:**
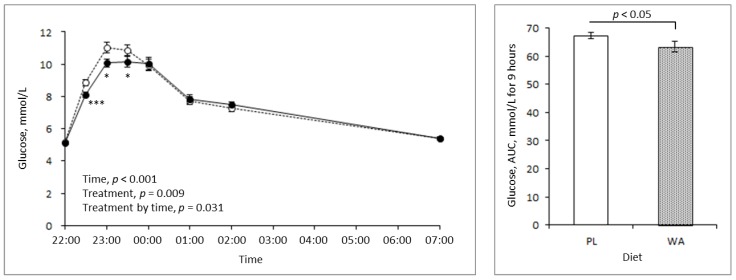
Changes in the blood glucose level during the night following the placebo (PL) (broken line, *n* = 20) and wheat albumin (WA) treatments (solid line, *n* = 20). (**Left**) Changes in the blood glucose response. (**Right**) The AUC of the blood glucose level for 9 h from 22:00 hour to 7:00 hour. Data are the mean ± standard error. Significant differences between the treatments: * *p* < 0.05, *** *p* < 0.001.

A comparison between the PL and WA treatments revealed that the increase in blood glucose concentration during the night was suppressed significantly by WA, as shown by the AUC. In the exploratory analysis, the treatment by time interaction was also shown to be significant. WA only suppressed the glucose response for 2 hours after the meal, but not after bedtime, suggesting that a hypoglycemic risk by WA treatment was not observed (Figure 2).

**Figure 3:**
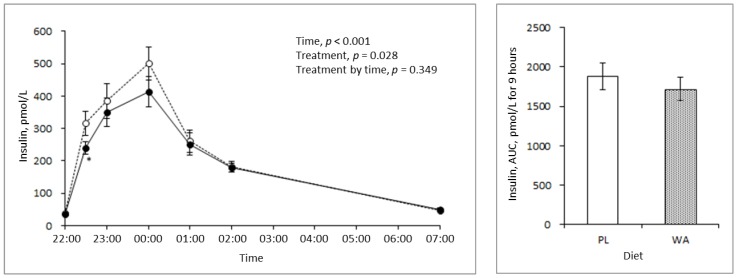
Changes in the blood insulin level during the night between the PL (broken line, *n* = 20) and the WA treatments (solid line, *n* = 20). (**Left**) Changes in the blood insulin response. (**Right**) The AUC of the blood insulin level for 9 h from 22:00 hour to 7:00 hour. Data are the mean ± standard error. Significant differences between the treatments: * *p* < 0.05.

Insulin response was not shown to be significantly different in the AUC and interaction assessments, however, a significant treatment effect was observed (Figure 3).

Blood triglyceride was also measured during the night, but no considerable differences between the treatments were observed (data not shown).

### 3.3. Effects of WA on Incretins during the Night

**Figure 4:**
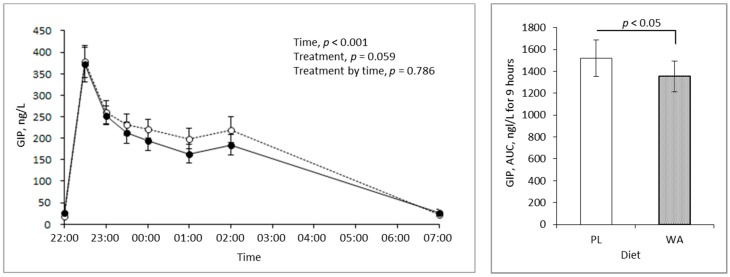
Changes in the blood glucose-dependent insulinotropic polypeptide (GIP) level during the night between the PL (broken line, *n* = 20) and WA treatments (solid line, *n* = 20). (**Left**) Changes in the blood GIP response. (**Right**) The AUC of the blood GIP level for 9 h from 22:00 hour to 7:00 hour. Data are the mean ± standard error.

**Figure 5:**
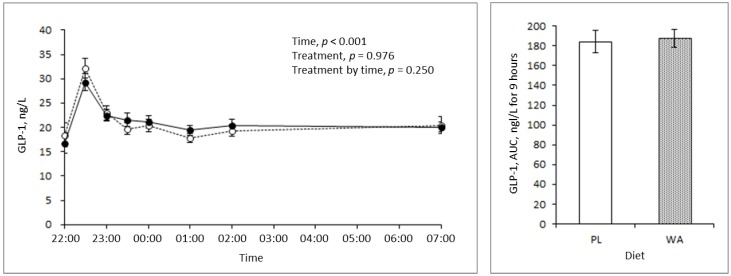
Changes in the blood active glucagon-like peptide-1 (GLP-1) level during the night between the PL (broken line, *n* = 20) and the WA treatments (solid line, *n* = 20). (**Left**) Changes in the blood GLP-1 response. (**Right**) The AUC of the blood GLP-1 level for 9 h from 22:00 hour to 7:00 hour. Data are the mean ± standard error.

The AUC of the GIP concentration in the blood was significantly lower following the WA treatment than following the PL treatment, but the treatment by time interaction was unchanged. Interestingly, the difference was large at bedtime, but there was no difference in the postprandial state for 2 hours after the meal (Figure 4). There were no significant differences in the blood GLP-1 concentration in any of the assessments (Figure 5).

## 4. Discussion

For healthy individuals, the recommended strategies to prevent an elevated glucose level at night are non-pharmaceutical, such as diet and exercise. Dietary therapy is perhaps easier to achieve, and thus may give more continuous efficacy, especially before sleep. Accompanied by the widespread use of a non-invasive glucose monitoring system, nocturnal hypoglycemia and its mortuary risk have been investigated in diabetic patients treated with insulin therapy [22]. However, in healthy individuals and individuals with borderline high values, the risk of a nocturnal hypoglycemic state is also best avoided. Therefore, the use of a food constituent with a moderate efficacy may be an alternative strategy for night care. As previously reported, WA has a suppressive effect on the postprandial glucose level via its inhibitory action on alpha-amylase activity [17], suggesting its potential as a low risk strategy for glycemic control during the night. Therefore, this study investigated the effect of WA on glucose response during the night in healthy individuals. As expected, WA contributed to a lower glucose response during the night but showed no hypoglycemic effect when compared to the PL (Figure 2). Thus, WA is a good candidate as a strategy for bedtime glycemic control in healthy individuals, and perhaps in diabetic patients alongside insulin therapy; however, additional studies are required for these patients to assess safety concerns.

An epidemiology study showed that the habitual intake of late-evening meals was associated with a higher BMI and metabolic risk factors such as high triglycerides and lower high-density lipoprotein cholesterol [23]. A potential explanation for this is the reduced metabolic rate and fuel utilization that occur during sleep [24]. GIP, an incretin that stimulates insulin secretion from pancreatic beta-cells, was reported to have a higher concentration postprandially that was directly associated with a lower metabolic rate [25] and the stimulation of fat accumulation in adipose tissue as an exopancreatic function. In contrast, GLP-1 did not show associations with these factors [26,27]. Moreover, higher blood GIP levels induced by chronic GIP treatment reduced fat utilization in high-fat diet-fed mice [28]. Interestingly, a bigger difference in the total GIP response between the PL and the WA treatments occurred after sleep than before sleep (Figure 4), with a moderate effect on insulin (Figure 3). These findings indicate that WA affects the later phase of the blood GIP level or its secretion, rather than the earlier phase. This might induce metabolic differences in the extrapancreatic actions of GIP, as mentioned above. Therefore, WA might improve not only glucose metabolism, but also fat utilization as energy by lowering the extrapancreatic actions of GIP during sleep. However, further investigation of the effects of WA on metabolic rate or fuel utilization during the night is required.

Dietary intake in the late evening has been suggested to alter clock genes such as *Bmal1* and *Clock* [29]. *Bmal1* knockout mice showed a higher blood glucose concentration than a wild type [30,31]. Circadian mutant mice, and both *Clock* [32,33] and *Bmal1* [34] mutants, showed impaired glucose tolerance, reduced insulin secretion, and defects in the size and proliferation of pancreatic islets that worsened with age. Thus, habitual late-evening meals may induce impaired glucose tolerance and the development of diabetes. As shown in our study, WA has the potential to reduce factors relating to the development of metabolic disorders, such as higher glucose and GIP responses during the night, through consumption in an evening meal, so WA can be expected to rearrange disordered circadian rhythms. Thus, a further investigation into the effect of WA on glucose tolerance with a focus on circadian rhythms or clock gene expression would be of great interest.

Overall, further studies to investigate the effects of WA on glycemic control during the night in diabetic patients, on energy metabolism, and on circadian clock genes would be of considerable interest. 

The limitations and potential biases in this study were the imbalanced gender of the participants (only men), the use of a single race (Japanese), and the inadequate sample size for stratified analyses based on the participants’ characteristics, such as BMI and fasting glucose level. In addition, all authors in this study are employees of the manufacturer of the studied ingredient.

## 5. Conclusions

WA might be a useful food constituent for glycemic control during the night.

## Figures and Tables

**Table 1:** Baseline characteristics of participants.

Parameter	Value
Number of participants (male/female)	20 (20/0)
Age, years	51 ± 1
Body weight, kg	75.8 ± 1.7
Body mass index, kg/m^2^	26.0 ± 0.4
Systolic blood pressure, mmHg	116 ± 2
Diastolic blood pressure, mmHg	76 ± 2
Glucose, mmol/L	5.19 ± 0.05
Insulin, pmol/L	39 ± 4
Triglyceride, mg/dL	1.61 ± 0.16
HbA1c, %	5.6 ± 0.1

Data are mean ± standard error.
